# Combined Serum Biomarkers in Non-Invasive Diagnosis of Non-Alcoholic Steatohepatitis

**DOI:** 10.1371/journal.pone.0131664

**Published:** 2015-06-29

**Authors:** Mei Yang, Dongping Xu, Yuan Liu, Xiaodong Guo, Wenshu Li, Chaonan Guo, Hongping Zhang, Yinjie Gao, Yuanli Mao, Jingmin Zhao

**Affiliations:** 1 Department of Pathology and Hepatology, Beijing 302 Hospital, Beijing, China; 2 Institute of Infectious Diseases/Liver Failure Medical Center, Beijing 302 Hospital, Beijing, China; 3 Center for Clinical Trial, Beijing 302 Hospital, Beijing, China; 4 Center for Clinical Laboratory, Beijing 302 Hospital, Beijing, China; Taipei Veterans General Hospital, TAIWAN

## Abstract

**Background:**

Non-alcoholic steatoheaptitis (NASH), the critical stage of non-alcoholic fatty liver disease (NAFLD), is of chronic progression and can develop cirrhosis even hepatocellular carcinoma (HCC). However, non-invasive biomarkers for NASH diagnosis remain poorly applied in clinical practice. Our study aims at testing the accuracy of the combination of cytokeratin-18 M30 fragment (CK-18-M30), fibroblast growth factor 21 (FGF-21), interleukin 1 receptor antagonist (IL-1Ra), pigment epithelium-derived factor (PEDF) and osteoprotegerin (OPG) in diagnosing NAFLD and NASH.

**Methods:**

179 patients with biopsy-proven NAFLD were enrolled as training group, 91 age- and gender-matched healthy subjects were recruited at the same time as controls. 63 other NAFLD patients were separately collected as validation group. 45 alcoholic fatty liver disease (AFLD) patients, 50 hepatitis B virus (HBV) patients, 52 hepatitis C virus (HCV) patients were also included. Serum biomarker levels were measured by enzyme-linked immunosorbent assay.

**Results:**

Serum levels of CK-18-M30, FGF-21, IL-1Ra and PEDF increased, while OPG decreased in a stepwise fashion in controls, non-NASH NAFLD patients and NASH patients (*P* < 0.01). The area under receiver-operating characteristics curve to diagnose NASH was 0.86 for CK-18-M30, 0.89 for FGF-21, 0.89 for IL-1Ra, 0.89 for PEDF and 0.89 for OPG. CK-18-M30 had 70% negative predictive value (NPV) and 79% positive predictive value (PPV) to diagnose NASH. A 5-step approach measuring CK-18-M30 followed by FGF21, IL-1Ra, PEDF and OPG gradually improved the NPV to 76% and PPV to 85%, which reached 80% and 76% respectively in the validation cohort.

**Conclusion:**

Compared to single biomarker, stepwise combination of CK-18-M30, FGF-21, IL-1Ra, PEDF and OPG can further improve the accuracy in diagnosing NASH.

## Introduction

Non-alcoholic fatty liver disease (NAFLD) has become the comonest form of chronic liver disease in European and Americn populations [1–3]. Recent studies show that the prevalence of NAFLD in adult population is 30–40% in the United States [4], 11–45% in northern Asia and 9–45% in South Asia as well as Southeast Asia during the past decade, and theses percentages continue to grow with the increase of obesity population worldwide [5]. NAFLD patients are more likely to suffer from liver fibrosis, liver cancer, type-2 diabetes, cardiovascular diseases, chronic glomerulo nephritis and other diseases [6–8]. NAFLD has become one of the most important public health issues in 21st century.

NAFLD includes a spectrum of diseases from simple fatty liver disease, to non-alcoholic steatohepatitis (NASH), liver cirrhosis and hepatocellular carcinoma (HCC). NASH, the critical stage in NAFLD progression, is the only bridge between NAFLD and liver cirrhosis [9–12], and its persistent existence has become one of the leading causes of liver cirrhosis and even HCC [13]. Statistical data show that 15%-20% of NASH patients will develop liver cirrhosis in 10 to 20 years [14–15], suggesting the risk of disease progression is much higher. Therefore, clinical diagnosis of NASH has gained more importance.

Although liver biopsy remains the ‘Gold Standard’ for the diagnosis of NASH [16], it is invasive and not easily accepted by patients. Non-invasive diagnosis by serum biomarkers are eagerly needed. Previous studies demonstrated that several serum biomarkers exhibit potential in diagnosing NAFLD and NASH. Cytokeratin-18 M30 fragment (CK-18-M30) could indicate hepatocyte apoptosis in NASH and was closely associated with NASH inflammation and fibrosis stage [17–21]. Fibroblast growth factor 21 (FGF-21) had the function of reducing blood glucose, lipid and insulin levels, reversing liver steatosis and improving insulin sensitivity [22, 23]. Interleukin 1 receptor antagonist (IL-1Ra), the only natural antagonist in cytokines, could suppress the activity of IL-1 and relieve the inflammation, pimelosis and damage of liver [24]. Pigment epithelium-derived factor (PEDF) is an independent risk factor of NAFL [25–27]. PEDF had the ability of anti-fibrosis and was closely related to the development and progression of fibrosis [28]. Osteoprotegerin (OPG) was closely associated with NASH steatosis and the OPG level in NASH patients was clearly lower than that in healthy subjects [29].

Neverthless, the feasibility and accuracy of these biomarkers have not been tested in large corhot, and the sensativity and specificity of the single biomarker in diagnosing NASH are relatively low according to previous reports. Moreover, single serum biomarker can only reflect one aspect of disease progression instead of the overall situation [30, 31]. Therefore, it raises the question whether the combination of these serum biomarkers could improve the accuracy in diagnosing NASH and what form of combination could achieve better diagnostic accuracy.

Our study aims at testing the accuracy of CK-18-M30, FGF-21, IL-1Ra, PEDF and OPG in non-invasive diagnosis of NAFLD and NASH. Meanwhile, our study will for the first time, combine these biomarkers and investigate what kind of combination could yield better performance in diagnosing NASH.

## Materials and Methods

### Study Participants

179 patients with biopsy-proven NAFLD from 2006 to 2013 were enrolled as training group, and 91 age- and gender-matched healthy subjects were recruited at the same time as controls. 45 alcoholic fatty liver disease (AFLD) patients, 50 hepatitis B virus (HBV) patients, 52 hepatitis C virus (HCV) patients were also included to differentiate NAFLD from other liver diseases. 63 other NAFLD patients were separately collected to validate the diagnosis efficacy. All patients have undergone liver biopsy, the criterion standard, and clinical examinations to ensure definite diagnosis of the disease.

Consecutive adult patients who underwent liver biopsy for suspected NAFLD at Beijing 302 Hospital, Beijing, China were retrospectively and prospectively screened into this study. Patients with history of excessive acloholic consumption (more than 140 g/week for male and 70 g/week for female; Acloholic Consumption = Intake volume × Alcoholic Strength × 0.8) [32] were excluded. Patients with liver diseases which can influence serum biomarker level including viral hepatitis, drug-induced liver disease, total parenteral nutrition, hepatolenticular degeneration and primary liver cancer were also excluded. Histological changes of the enrolled subjects should meet the pathological criteria for fatty liver diseases.

Age (±2 yrs) and gender-matched healthy cases from physical examination population also at Beijing 302 Hospital, Beijing, China were recruited at the same time as controls. All these cases had passed comprehensive liver and metabolic assessment and the recruitment standards included: 1) in good health; 2) with normal liver function; 3) B-ultrasound examination showed no symptoms of fatty liver; 4) without history of excessive alcoholic consumption; 5) without history of diabetes, hypertension and normal metabolic parameters.

All AFLD, HBV and HCV cases had clear evidence of liver injury based on pathological diagnosis and clinical examinations made according to clinical practice guidelines [33–35]. Other enrollment criteria were as follows: 1) history of excessive alcoholic consumption (>140 g/week for male and 70 g/week for female) for AFLD group; 2) Clinical history of chronic hepatitis B or HBsAg (+) for more than 6 months for HBV cases; 3) Clinical history of chronic hepatitis C and no HCV/HIV co-infection for HCV group; 4) The biochemical indicators of the enrolled cases have met with the specific standard of each liver disease. All these three groups of patients should have no history of HCC, liver transplant or decompensated liver diseases.

The study was conducted according to the guidelines of the Declaration of Helsinki. The protocol was approved by the Ethics Committee of Beijing 302 Hospital and all subjects gave their written informed consent to participate in this study.

### Clinical and Biochemical Characterization

All subjects were given physical examination as well as physiological and biochemical detection. Body mass index (BMI) was calculated by measurement of height and weight (BMI = weight (kg) / height^2^ (m^2^)).

Fasting venous blood of all subjects (NAFLD, AFLD, HBV and HCV patients one day before the liver biopsy) were collected to evaluate liver function and related biochemical indicators, including aspartate aminotransferase (AST), alanine aminotransferase (ALT), AST / ALT, γ-glutamyl transpeptidase (GGT), alkaline phosphatase (ALP), cholinesterase, total cholesterol (TC), triglyceride (TG), high density lipoprotein (HDL), low-density lipoprotein (LDL).

### Liver Histology

Percutaneous liver biopsy was performed by experienced physicians with a 16-gauge Hepafix needle under the guidance of B- ultrasound [36]. The liver biopsy specimens were immediately fixed in 10% formalin and embedded in paraffin. The histological slides were read and semi-quantitatively scored by two experienced pathologists. Histological grading and staging of NAFLD were scored according to NAFLD activity score (NAS), a semi-quantitative scoring system reported by Kleiner and his colleagues [37]. NAS, ranging from 0–8, was the sum of steatosis, lobular inflammation and hepatocellular ballooning scores. Steatosis was scored from 0 to 3: S0: no steatosis or less than 5%, S1: 5–33%, S2: 34–66%, S3: >66%. Lobular inflammation was graded as follows: stage 0, no foci; stage 1: < 2 foci per 200 × field; stage 2: 2–4 foci per 200 × field; stage 3: > 4 foci per 200 × field. Ballooning degeneration of liver cells was evaluated as: grade 0, absent; grade 1, few; grade 2, a lot. For analysis in this study, NAFLD cases with NAS≥5 were considered as NASH patients and NAFLD cases with NAS≤4 were grouped as non-NASH patients including simple fatty liver cases (NAS 0–2) and borderline NASH cases (NAS 3–4) [38, 39].

### Serum CK-18-M30, FGF-21, IL-1Ra, PEDF and OPG

3–4 ml antecubital vein blood was collected from each subject after an overnight fasting. Blood samples were centrifuged at 3000 r/min for 10 min and serum aliquots were stored at −80°C for detection. Serum biomarkers levels were quantified by commercially available ELISA kits (Human CK-18-M30, FGF-21, IL-1Ra, PEDF, OPG, R&D, USA) according to the manufacturer's protocol.

### Statistical Analysis

Continuous variables were expressed as mean ± standard deviation. Clinical data between groups were compared by Chi-squared test; quantitative variables were analyzed using t-test and one-way analysis of variance for normal distributional data. Intergroup comparisons were performed using ANOVA. Spearman’s correlation coefficient was used to estimate the correlation between serum biomarkers levels and related factors. LSD test was used for multiple pairwise comparison. Multiple linear regression was used to determine the independent factors associated with biomarker levels. Receiver operating characteristic (ROC) curve analysis was conducted to assess the performance of biomarkers in the diagnosis of NAFLD/NASH by calculating the area under receiver operating characteristic curve (AUROC). The maximum sensitivity and specificity of biomarker combination were determined by Youden index. Rank correlation was used to handle variables that were not normally distributed. The statistics were analyzed using the SPSS 18.0 for windows (SPSS, Inc, Chicago, IL, USA) and SAS 9.0 (SAS, Inc, Cary NC, USA). A two-tailed *p* value of < 0.05 showed the differences were statistically significant.

## Results

### Patients’ Characteristics

179 NAFLD patients in training group included 86 males and 93 females with a mean age of 30.3±11.8 yrs. Among them, 52 had NAS 0–2, 59 had NAS 3–4 and 68 had NAS≥5, making a 68 NASH cases and 111 non-NASH cases. The validation group was consisted of 33 males and 30 females (mean age, 31.6±9.2yrs), including 20 NAS 0–2, 18 NAS3-4 and 25 NAS≥5. 30–45% of the NAFLD patients were accompanied with type 2 diabetes and hypertension. No significant differences existed in the clinical biochemical indicators between training group and validation group. 91 healthy controls (42 males, 49 females), 45 AFLD patients (24 males, 21 females), 50 HBV patients (29 males, 21 female) and 52 HCV patients (23 males, 29 females) were also enrolled at the same time.

All groups did not differ in terms of age and gender. The disease groups (NAFLD, AFLD, HBV and HCV) and healthy cases had significant differences in clinical biochemical indexes (Table 1). Significant differences existed in the levels of ALT, AST, TG and BMI between non-NASH and NASH cases (*P* < 0.01). NAFLD group, HBV group and HCV group had differences in TC and TG (*P* < 0.05) and significant difference in AST/ALT (*P* < 0.01). Compared with other diseases, cholinesterase was obviously higher in NAFLD *(P* < 0.01). BMI levels showed obvious differences among NAFLD, HBV and HCV group, but not between NAFLD and AFLD cases.

**Table 1:** Clinical characteristics of all subjects.

	Control	NAS0~2	NAS3~4	NASH	AFLD	HBV	HCV
**All**	91	52	59	68	45	50	52
**Age (yr)**	29.40±7.00	28.30±9.70	30.90±14.00	31.70±13.50	38.00±7.52	30.36±15.51	35.33±19.31
**Gender (male/female)**	42/49	29/23	26/33	31/37	24/21	29/21	23/29
**BMI (kg/m** ^**2**^ )**	19.80±2.10	25.60±2.90	26.90±3.60	28.70±3.60	26.74±1.66	22.11±4.39	20.37±2.06
ALT(U/L)**	27.20±10.30	65.90±26.10	108.80±36.70	160.60±28.50	122.88±120.51	81.55±67.09	48.67±51.00
AST(U/L)**	29.60±5.90	33.80±16.50	50.10±20.30	89.60±25.20	47.63±28.55	87.55±88.24	52.00±44.13
**AST/ALT$$**	0.89±0.26	0.53±0.15	0.65±0.18	0.64±0.21	0.60±0.33	1.29±1.24	1.26±0.49
**ALP(U/L)**	93.30±16.20	100.80±18.60	115.40±17.30	157.50±13.70	96.00±23.53	129.55±94.80	117.78±106.18
**GGT(U/L)**	36.30±17.60	150.30±20.50	90.10±16.70	87.50±18.60	63.88±53.19	69.91±152.10	30.67±21.12
**Cholinesterase (U/L)**	9025.80 ±1231.20	8651.40 ±1253.20	9239.00 ±1478.20	9742.80 ±1576.30	8522.89 ±2279.93	6576.40 ±1516.10	7303.89 ±1411.10
**TC(mmol/L)##**	4.75±0.86	4.87±0.94	4.69±0.78	4.78±1.13	4.43±0.84	3.78±0.42	3.61±0.92
**TG(mmol/L)##**	1.83±0.49	2.31±0.67	2.01±0.56	1.95±0.36	1.79±0.68	1.35±1.34	1.03±0.35
**HDL(mmol/L)**	0.91±0.34	1.06±0.22	0.95±0.17	1.06±0.25	0.95±0.13	1.19±0.32	1.17±0.27
**LDL(mmol/L)**	2.88±0.31	3.61±0.67	3.35±0.63	3.24±0.59	3.10±0.64	2.55±0.40	2.36±0.62

NAFLD, AFLD, HBV, HCV patients and age-and gender-matched control subjects were enrolled from 2006 to 2013. Significant differences existed in the levels of ALT, AST, TG and BMI between non-NASH and NASH cases.

** Between Non-NASH and NASH groups, significant at *P* < 0.01.

^##^ Among NAFLD, HBV, HCV group, significant at *P* < 0.05.

^$$^ Among NAFLD, HBV, HCV group, significant at *P* < 0.01

All NASH patients had steatosis and lobular inflammation, while 89.71% of them (n = 61) had ballooning and 92.65% (n = 63) had fibrosis. Significant differences existed in histological features of NAFLD, such as steatosis, ballooning, fibrosis and lobular inflammation, between non-NASH and NASH groups. Steatosis, lobular inflammation, balloon degeneration of hepatocytes, and fibrosis in NASH patients were more serious than those of non-NASH cases (*P* < 0.01) (S1 Table).

### Levels of Serum Biomarkers

**Fig 1:**
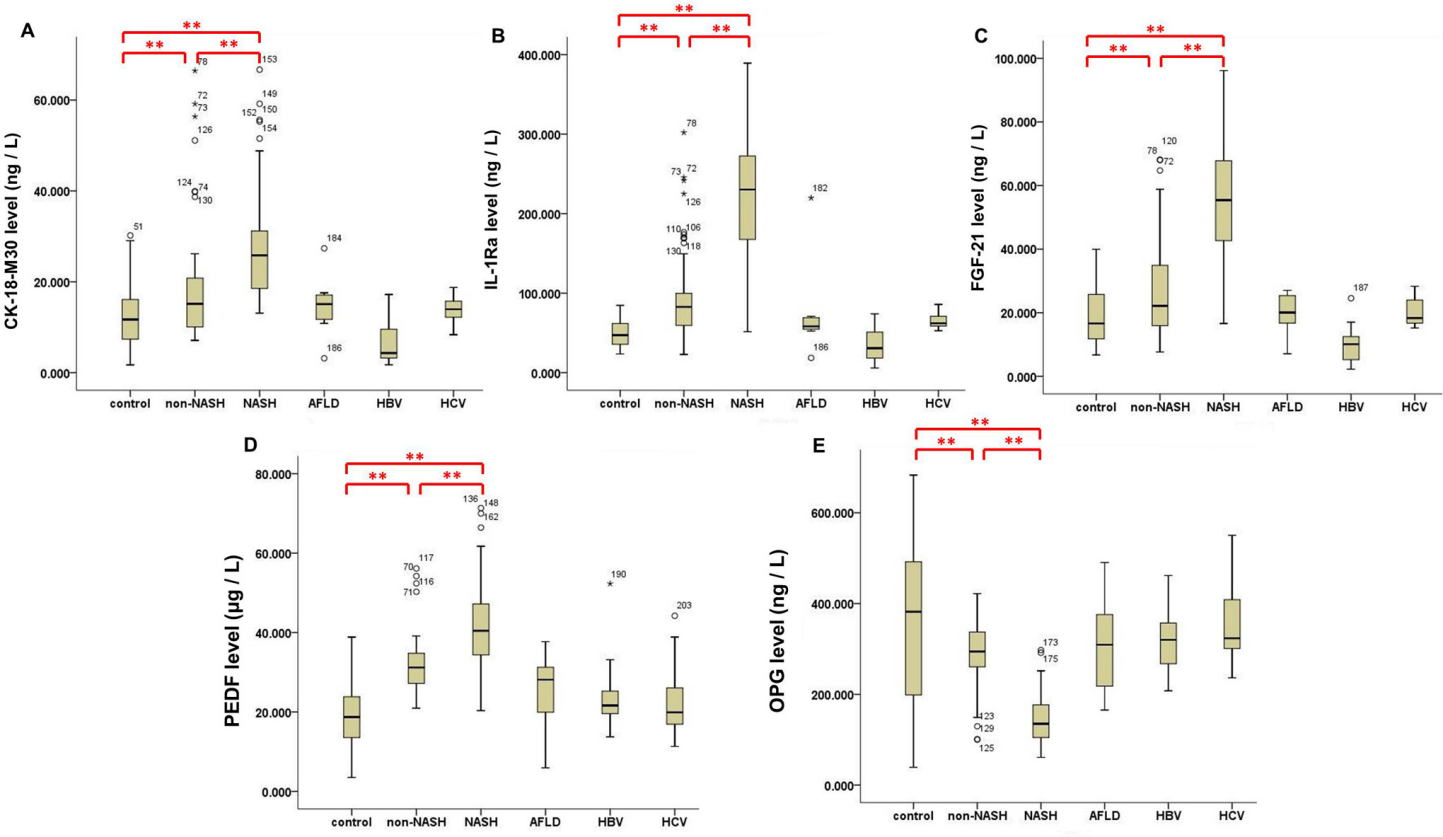
Serum levels of five biomarkers in control, non-NASH, NASH and other disease groups. Levels of CK-18-M30, FGF-21, IL-1Ra and PEDF were significantly higher in NAFLD training group (A-D) whereas OPG was significantly lower (E), significant at **P < 0.01. There were no statistically significant differences among the serum biomarker levels between healthy controls and AFLD, HBV and HCV groups.

After analyzing the serum biomarker levels in training group and controls, we found that CK-18-M30, IL-1Ra, FGF-21 and PEDF were significantly higher in NAFLD training group (median 18.30 ng/L, 104.80 ng/L, 39.30 ng/L, and 33.50 μg/L respectively) than controls (median 14.10 ng/L, 47.10 ng/L, 16.60 ng/L and 18.70 μg/L respectively). On the contrary, OPG was significantly lower in training group (median 234.10 ng/L) than in controls (median 381.90 ng/L). There were no statistically significant differences among the serum levels of CK-18-M30, IL-1Ra, FGF-21, PEDF and OPG between healthy controls and AFLD, HBV and HCV groups (*P* > 0.05) (Fig 1; S2 Table).

Also the levels of CK-18-M30, IL-1Ra, FGF-21 and PEDF in NAFLD cases in the training group increased with the elevation of NAS scores while OPG level decreased conversely (S1 Fig). Interestingly, we noted that serum level of some biomarkers specifically differed in certain groups. For example, Serum OPG level could distinguish simple fatty liver patients form the controls (*P* = 0.01). Serum PEDF level in the borderline NASH patients significantly differed from other NAFLD cases (*P* < 0.05). Borderline NASH patients and simple fatty liver patients were distinguished significantly by PEDF level. All serum biomarkers levels were highly correlated with NAS (*P* < 0.01) (S3 Table).

### Correlation between Serum and Clinical Biomarkers in Diagnosing NASH

Serum levels of CK-18-M30, IL-1Ra, FGF-21, PEDF and OPG were highly correlated with clinical characteristics and pathological features (S3 Table). Multiple linear regression analysis was conducted to study the association between serum biomarkers levels and pathological features of NASH in NAFLD patients after adjusting for age and gender. We found that steatosis, severe lobular inflammation, ballooning and fibrosis were independent factors of all serum biomarkers.

### ROC of Serum Biomarkers in Diagnosing NASH

To test the effectiveness of these serum biomarkers in diagnosing NASH, we performed ROC curve analysis on both non-NASH and NASH cases. All five biomarkers showed good diagnostic performance, and the AUROC were calculated to determine best cut-off value, sensitivity and specificity of each biomarker in diagnosing NASH (Table 2, S2 Fig).

**Table 2:** AUROC and OR analysis of serum biomarkers to determine effectiveness in diagnosing NASH.

Biomarker	AUROC	Cut-off Value	Sensitivity	Specificity	OR	95% Confidence Interval	
CK-18-M30	0.86	17.75 ng/L	80.30%	79.60%	2.25	0.68	7.49
IL-1Ra	0.89	98.47 ng/L	80.10%	76.30%	10.06	2.92	34.69
FGF-21	0.89	40.64 ng/L	79.30%	77.40%	24.76	6.82	89.86
PEDF	0.89	23.96 μg/L	79.60%	78.40%	1.21	0.37	3.94
OPG	0.89	252.18 ng/L	81.30%	74.60%	1.38	0.36	5.26

AUROC were calculated to determine best cut-off value, sensitivity and specificity of serum CK-18-M30, IL-1Ra, FGF-21, PEDF and OPG in diagnosing NASH.Logistic regression analysis was performed by using cut-off value of serum biomarkers in diagnosing NASH. OR of each serum biomarker and 95% confidence intervals were also determined.

### Multivariate Logistic Regression Analysis

After adjusting some confounding factors such as age and gender, we performed Logistic regression analysis using cut-off value of each biomarker in NASH diagnosis, and obtained the following two regression equations.

The first one involved only the five biomarkers:

Logistic Y1=−1.77+1.16CK−18−M30+3.20FGF−21+2.57IL−1Ra+1.80OPG−2.06PFDF

And the second one was added other clinical biochemical indicators:

Logistic Y2=−0.65+0.23CK−18−M30+0.36FGF−21+3.15OPG−2.75PEDF+2.37BMI+0.45ALT+0.11AST+1.02TG+0.37HDL+0.61LDL+3.78Cholinesterase.

Relative risk (OR) of each serum biomarker and 95% confidence intervals (95% CI) were also determined (Table 2).

### Diagnostic Accuracy of Logistic Models

The diagnostic performance of Logistic models was firstly evaluated in training group by ROC curves. When Logistic Y1 was applied, the AUROC of NASH diagnosis was 0.53. Under the condition of maximum sensibility and specificity, the cut-off value and 95% CI were 1.94 and 0.24–0.36, respectively. The diagnostic sensibility and specificity also reached 43% and 56%. For Logistic Y2, the AUROC was 0.44, and the sensibility and specificity for diagnosis was 38% and 44%, respectively.

Then we accessed the diagnostic performances of two Logistic models in validation group. The AUROC was 0.56 for Logistic Y1, while the sensibility and specificity were 42% and 51%, respectively. For Logistic Y2, the AUROC was 0.48, and the sensibility and specificity for diagnosis was 40% and 46%, respectively. There was no statistical differences between training group and validation group *(P > 0*.*05*) (S4 Table).

### 5-step Layered Combination of Serum Biomarkers in Diagnosing NASH

Since the regression equation did not demonstrate better effect in diagnosing NASH than single biomarker did, we decided to use another form of biomarker combination, a 5-step approach measuring CK-18-M30 successively followed by FGF21, IL-1Ra, PEDF and OPG. When FGF21, IL-1Ra, PEDF and OPG were added in proper sequence on the basis of CK-18-M30, the negative predictive value (NPV) and positive predictive value (PPV) in diagnosing NASH both gradually increased (Table 3).

**Table 3:** Stepwise combination of serum biomarkers increased diagnosis performance for NASH in NAFLD patients.

	Sensitivity (%)	Specificity (%)	PPV (%)	NPV (%)
**CK-18-M30**	87.00	90.00	79.00	71.00
**IL-1Ra**	84.00	91.00	80.00	73.00
**FGF-21**	79.00	88.00	84.00	69.00
**PEDF**	83.00	75.00	78.00	70.00
**OPG**	85.00	82.00	83.00	64.00
**Combined Model**	94.00	96.00	85.00	76.00

Combined model demonstrated better effect in diagnosing NASH than single serum biomarker.

Combined Model: Stepwise combination of CK-18-M30, IL-1Ra, FGF-21, PEDF, and OPG in NASH diagnosis.

**Fig 2:**
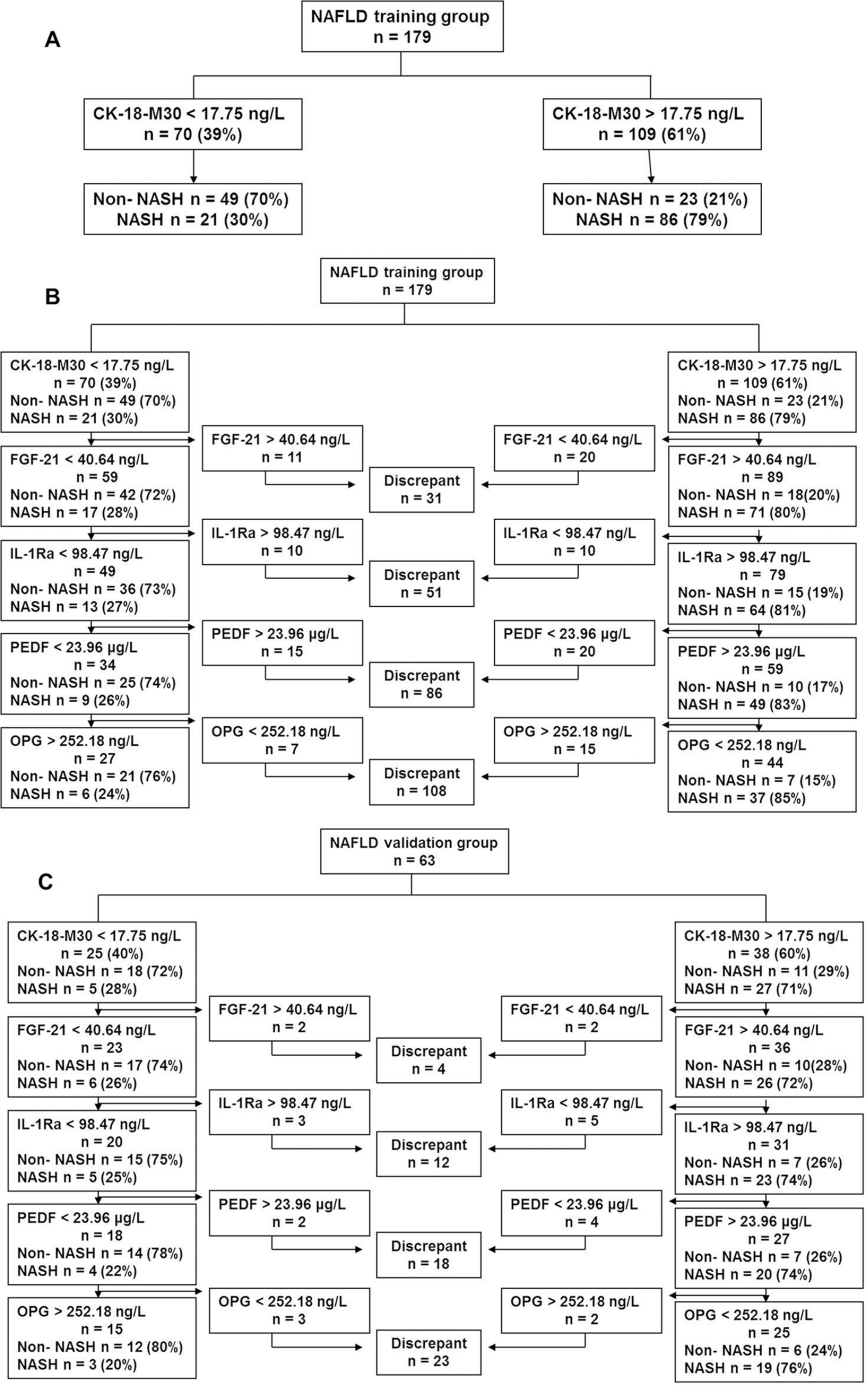
NASH diagnosis by serum biomarkers in NAFLD training group and validation group. (A) NASH diagnosis solely by CK-18-M30. (B) 5-step combination of serum biomarkers in diagnosing NASH in training group. (C) 5-step combination of serum biomarkers in diagnosing NASH in validation group.

When CK-18-M30 was used alone, 70 patients (39%) had a CK-18-M30 level below 17.75 ng/L. 49 of these 70 patients were non-NASH patients, yielding a NPV of 70% and a sensitivity of 87%. In contrast, 109 subjects had a CK-18-M30 level above 17.75 ng/L, in which 86 subjects were NASH patients, yielding a PPV of 79% and a specificity of 90% (Fig 2A).

FGF-21, IL-1Ra, PEDF and OPG were successively added and the accuracy in diagnosing NASH was further improved. Among the 70 patients with CK-18-M30 level below 17.75 ng/L, 59 had a FGF21 level below 40.64 ng/L. 42 patients did not have NASH, yielding a NPV of 72% and a sensitivity of 88%. Among 109 patients with CK-18-M30 level above 17.75 ng/L, 89 had FGF-21 above 40.64 ng/L, yielding a PPV of 80% and a specificity of 91% (Fig 2B). Finally, when IL-1Ra (cut-off valve 98.47 ng/L), PEDF (cut-off value 23.96 μg/L) and OPG (cut-off value of 252.18 ng/L) were added sequentially, the NPV was elevated to 76%, sensitivity to 94%, PPV to 85%, and specificity to 96% (Fig 2B).

The 5-step approach was then validated in 63 NAFLD patients with paired liver biopsies, in which the proportion of NASH patients was similar to that in training group. 15 out of the 63 validation cases had twice follow–up biopsies and the comparison of two biopsy results showed the 15 cases had progressed from non-NASH to NASH. In these prospective follow–up cases, levels of serum CK-18-M30, FGF-21, IL-1Ra and PEDF were increased, while OPG decreased in cases in non-NASH stage (first biopsy) compared to NASH stage (second biopsy). In 63 NAFLD validation patients, 25 cases had a CK-18-M30 level below the cut-off value, and 38 cases had a CK-18-M30 level above the cut-off value. Then we continued to diagnose NASH with FGF-21, IL-1Ra, PEDF and OPG on this basis, and found 12 patients were non-NASH and the NPV was up to 80%, sensitivity to 95%. 19 NASH cases were predicted by the established noninvasive diagnostic models, the PPV was 76% and the specificity was 97% (Fig 2C).

## Discussion

Previous studies demonstrated that the serum levels of CK-18-M30, FGF-21, IL-1Ra, PEDF and OPG exhibited significant differences in patients with NAFLD [40–43, 26]. Growing evidence suggest that these markers play an important role in the pathogenesis of NAFLD. Meanwhile, these markers involve in NAFLD development and are related to steatosis, inflammation, hepatocyte apoptosis and fibrosis during disease progression [44–51]. Our study showed the serum levels of CK-18-M30, FGF-21, IL-1Ra and PEDF were significantly higher in NAFLD patients compared with healthy subjects, positively correlated with NAS scores and pathological characteristics of NAFLD, while the serum level of OPG was the opposite. These findings and the previous reports showed these factors were correlated with NAFLD, leading to the preliminary conclusion that the five biomarkers could be noninvasive diagnostic markers for NAFLD.

To further clarify whether these biomarkers were specific to NAFLD or exhibited differences in all kinds of liver diseases, we tested the serum levels of the biomarkers in AFLD, HBV and HCV groups simultaneously. Interestingly, we found that the statistical differences between NAFLD and healthy controls were more significant than those of other diseases. This further confirmed our presupposition that the five biomarkers had higher specificity for NAFLD and potential in diagnosing NAFLD and NASH.

In addition, we also discovered that the levels of these biomarkers could serve as evaluation indicators of NAFLD degree. An interesting finding during our study was that some biomarkers were specifically differed in cases with certain degree of NAFLD, which meant that they could be used to differentiate different degrees of NAFLD. For example, OPG could differentiate control group and simple fatty liver patients, while PEDF could clearly distinguish simple liver patients from NASH patients. The serum levels of biomarkers in follow-up cases changed with disease progression from non-NASH to NASH. Thus, we could come to the first conclusion that CK-18-M30, FGF-21, IL-1Ra, PEDF and OPG were noninvasive diagnostic serum biomarkers for NAFLD and NASH.

The logistic regression analysis revealed these five biomarkers were independent risk factors of NASH progression. Population with abnormal levels of CK-18-M30, FGF-21, IL-1Ra, PEDF and OPG seemed more possible to suffer from NASH, indicating these biomarkers have a crucial place in NASH prediction.

Two noninvasive diagnosis models were established based on logistic regression analysis of the five serum biomarkers and the other clinical biochemical indicators. However, the ROC curve analysis of the two models in NASH diagnosis showed that the sensibility, specificity, PPV and NPV of two models were lower than those of single index. These findings were in accordance with the previous studies in which they found the diagnostic efficiency of the combined model might be lower than that of the single index. A group of Spanish researchers found that Forns index, a mathematic model, could only distinguish slight hepatic fibrosis, in contrast, the positive rate of diagnosis for significant hepatic fibrosis was lower [52]. The report indicated that this model couldn’t predict and diagnose for hepatic fibrosis accurately [52] and the ideal noninvasive model should possess the characteristics of high accuracy (sensibility, specificity, NPV and PPV values). Nevertheless, Shen *et al* [53] concluded that the 2-step combination of serum biomarkers could improve the sensibility and specificity of NASH diagnosis. Therefore, our study turned to try the stepwise combination of these five biomarkers for NASH diagnosis.

Combination of these five biomarkers in different sequences had been tried and we finally found the 5-step approach measuring CK-18-M30 successively followed by FGF21, IL-1Ra, PEDF and OPG could achieve better diagnostic performance. The NPV and PPV increased to 76% and 85% respectively and the noninvasive test efficiency was improved. Cases whose non-invasive diagnosis results were not consistent with the biopsy results were put into discrepant zones. The 5-step approach measuring biomarkers increased the noninvasive test efficiency compared to the partial combination of biomarkers. The validation experiment showed that this noninvasive step-by-step test exhibited good sensibility and accuracy in diagnosing NASH.

Our study has several limitations. Firstly, sampling bias may occur as liver biopsy was used as the gold standard. However, liver biopsy is currently the only reference standard for NASH diagnosis. Besides, the biopsy specimens were assessed by two experienced pathologists, which could eliminate the influence of human factors. Secondly, liver biopsies were not performed in control subjects. It is unethical to perform biopsy in cases without liver diseases. Instead, the selection of healthy population was based on physical examination, strict laboratory tests and B-ultrasonic examination, which could exclude the interference of other diseases. Thirdly, the validation cohort was an independently collected and could accurately evaluate the model. Fourthly, the samples were all selected from Chinese population, whether our results could be extrapolated to populations with different ethnic background needs further research. The limitations are common in some genetic and ethnically homogeneous researches.

In conclusion, the serum levels of CK-18-M30, FGF21, IL-1Ra, PEDF and OPG were strongly associated with NASH progression and may serve as biomarkers of NASH identification. The stepwise combination of these biomarkers could further enhance NASH diagnosis accuracy and played an important role in NAFLD clinical diagnosis, therapeutic effect evaluation as well as prevention.

## Supporting Information

S1 Fig Serum biomarker levels increased/decreased with NAS. **(TIF)** (A~D) The serum levels of CK-18-M30, IL-1Ra, FGF-21 and PEDF in NAFLD training group increased with the elevation of NAS scores. (E) The serum levels of OPG level decreased in NAFLD training group with the elevation of NAS score.
(TIF)

S2 Fig ROC of serum biomarkers in diagnosing NASH. **(TIF)** The five biomarkers showed good diagnostic performance for NASH. (A) NASH diagnosis by CK-18-M30. (B) NASH diagnosis by FGF-21. (C) NASH diagnosis by IL-1Ra. (D) NASH diagnosis by PEDF. (E) NASH diagnosis by OPG.
(TIF)

S1 Table Histological features of NAFLD patients. (S1 Table, DOC)
(DOC)

S2 Table Mean of serum biomarker levels among non-NASH, NASH, control and other disease groups. (S2 Table, DOC)
(DOC)

S3 Table Correlation between serum biomarkers and clinical biomarkers in NAFLD training group. (S3 Table, DOC)
(DOC)

S4 Table Diagnostic performances of Logistic models for NASH diagnosis in NAFLD training group and validation group. (S4 Table, DOC)
(DOC)
