# Supplementary figures and images for: Combined Serum Biomarkers in Non-Invasive Diagnosis of Non-Alcoholic Steatohepatitis
Source: PLoS One. 2015 Jun 29;10(6):e0131664. doi: 10.1371/journal.pone.0131664 (PMC4486729; doi:10.1371/journal.pone.0131664)

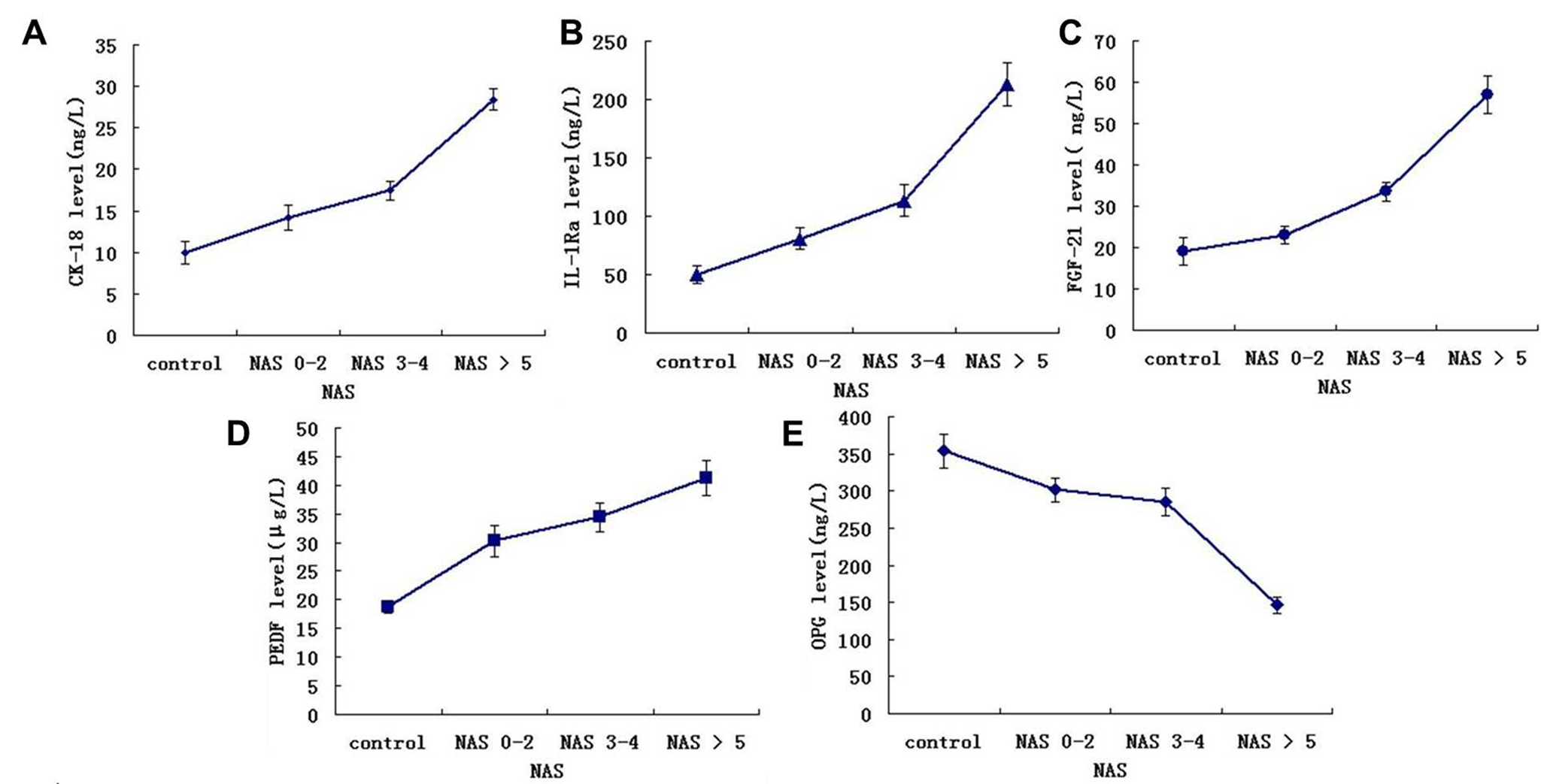

Supplement: S1 Fig — (TIF) (A~D) The serum levels of CK-18-M30, IL-1Ra, FGF-21 and PEDF in NAFLD training group increased with the elevation of NAS scores. (E) The serum levels of OPG level decreased in NAFLD training group with the elevation of NAS score. (TIF) [file pone.0131664.s001.tif]

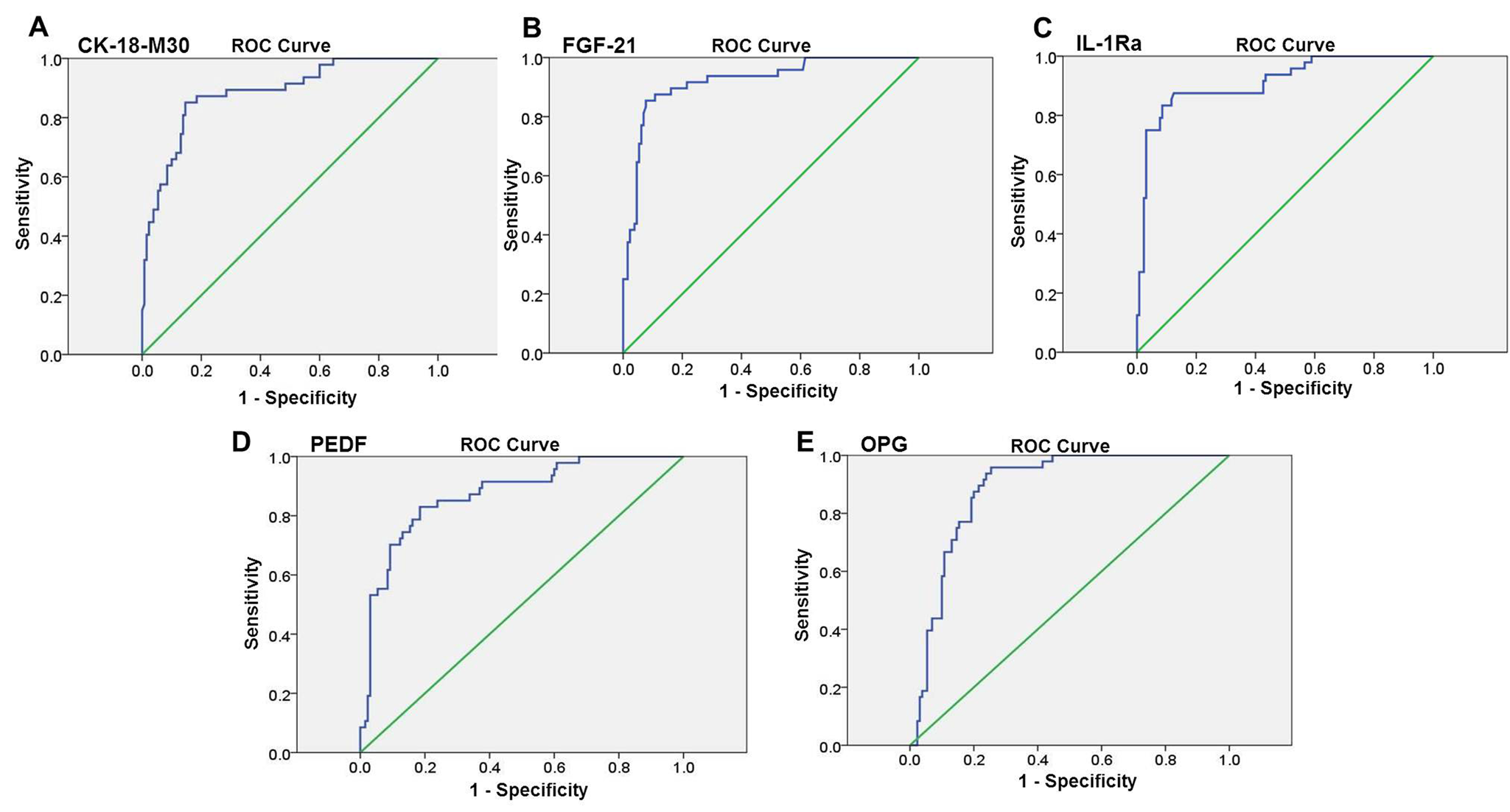

Supplement: S2 Fig — (TIF) The five biomarkers showed good diagnostic performance for NASH. (A) NASH diagnosis by CK-18-M30. (B) NASH diagnosis by FGF-21. (C) NASH diagnosis by IL-1Ra. (D) NASH diagnosis by PEDF. (E) NASH diagnosis by OPG. (TIF) [file pone.0131664.s002.tif]
