# Supplementary material for: Combined Serum Biomarkers in Non-Invasive Diagnosis of Non-Alcoholic Steatohepatitis
Source: PLoS One. 2015 Jun 29;10(6):e0131664. doi: 10.1371/journal.pone.0131664 (PMC4486729; doi:10.1371/journal.pone.0131664)
Supplement: S1 Table — (S1 Table, DOC) (DOC) [file pone.0131664.s003.doc]

**S1 Table. Histological features** of NAFLD patients.

|  | **Control** | **NAS0~2** | **NAS3~4** | **NASH** |
| --- | --- | --- | --- | --- |
| **All** | 91 | 52 | 59 | 68 |
| **Steatosis grade1/2/3**** | - | 41/11/0 | 6/39/14 | 0/11/57 |
| **Lobular inflammation 0/1/2/3**** | - | 13/39/0/0 | 16/43/0/0 | 0/45/14/9 |
| **Ballooing 0/1/2**** | - | 48/4/0 | 23/29/7 | 7/43/18 |
| **Fibrosis 0/1/2/3/4**** | - | 52/0/0/0/0 | 10/38/11/0/0 | 5/21/16/19/7 |
| **Periportal inflammation Mild / Moderate / Severe** | - | 52/0/0 | 46/13/0 | 43/11/14 |

NAFLD patients and control subjects were matched by gender and age (±2years).

** Between Non-NASH and NASH groups, significant at *P* < 0.01.
