# Supplementary material for: Combined Serum Biomarkers in Non-Invasive Diagnosis of Non-Alcoholic Steatohepatitis
Source: PLoS One. 2015 Jun 29;10(6):e0131664. doi: 10.1371/journal.pone.0131664 (PMC4486729; doi:10.1371/journal.pone.0131664)
Supplement: S2 Table — (S2 Table, DOC) (DOC) [file pone.0131664.s004.doc]

**S2 Table.** Mean of serum biomarker levels among non-NASH, NASH, control and other disease groups.

| **Biomarker  (mean)** | **control** | **Non-NASH** | **NASH** | **AFLD** | **HBV** | **HCV** |
| --- | --- | --- | --- | --- | --- | --- |
|  | **n = 91** | **n = 111** | **n = 68** | **n = 45** | **n = 50** | **n = 52** |
| CK-18-M30(ng/L) | 12.92 | 18.76 | 28.42 | 14.79 | 7.19 | 13.87 |
| IL-1Ra(ng/L) | 49.80 | 95.29 | 212.73 | 75.33 | 34.40 | 66.16 |
| FGF-21(ng/L) | 19.10 | 27.65 | 56.88 | 19.82 | 10.07 | 20.30 |
| PEDF(μg/L) | 18.73 | 32.09 | 41.28 | 25.28 | 24.49 | 23.56 |
| OPG(ng/L) | 353.70 | 294.09 | 146.76 | 307.75 | 321.61 | 349.45 |

Serum levels of CK-18-M30, IL-1Ra, FGF-21 and PEDF were significantly higher in NASH group than controls. However, OPG was significantly lower in NASH group than in controls. There were no statistically significant differences among the serum biomarkers between healthy controls and AFLD, HBV and HCV groups.
