# Supplementary material for: Combined Serum Biomarkers in Non-Invasive Diagnosis of Non-Alcoholic Steatohepatitis
Source: PLoS One. 2015 Jun 29;10(6):e0131664. doi: 10.1371/journal.pone.0131664 (PMC4486729; doi:10.1371/journal.pone.0131664)
Supplement: S3 Table — (S3 Table, DOC) (DOC) [file pone.0131664.s005.doc]

**S3 Table**. Correlation between serum biomarkers and clinical biomarkers in NAFLD training group.

|  | **CK-18-M30** | | **IL-1Ra** | | **FGF-21** | | **PEDF** | | **OPG** | |
| --- | --- | --- | --- | --- | --- | --- | --- | --- | --- | --- |
|  | **rho** | ***P*** | **rho** | ***P*** | **rho** | ***P*** | **rho** | ***P*** | **rho** | ***P*** |
| **Gender** | 0.25 | 0.10 | -0.22 | 0.01 | -0.16 | 0.051 | -0.39 | 0.14 | 0.04 | 0.67 |
| **Age** | -0.22 | 0.01 | -0.19 | 0.02 | -0.29 | 0.00 | -0.10 | 0.22 | 0.17 | 0.04 |
| **BMI** | 0.14 | 0.03 | 0.09 | 0.04 | 0.16 | 0.01 | 0.04 | 0.01 | -0.37 | 0.00 |
| **ALT** | -0.21 | 0.01 | 0.66 | 0.00 | 0.53 | 0.00 | 0.58 | 0.00 | -0.34 | 0.00 |
| **AST** | -0.15 | 0.08 | 0.67 | 0.00 | 0.57 | 0.00 | 0.58 | 0.00 | -0.32 | 0.00 |
| **ALP** | -0.09 | 0.42 | -0.03 | 0.79 | 0.06 | 0.61 | 0.07 | 0.54 | -0.10 | 0.39 |
| **GGT** | -0.23 | 0.01 | 0.58 | 0.00 | 0.45 | 0.00 | 0.56 | 0.00 | -0.28 | 0.00 |
| **TC** | -0.10 | 0.26 | 0.28 | 0.00 | 0.21 | 0.02 | 0.20 | 0.03 | -0.16 | 0.07 |
| **TG** | -0.43 | 0.00 | 0.39 | 0.00 | 0.26 | 0.00 | 0.47 | 0.00 | -0.09 | 0.32 |
| **HDL** | 0.31 | 0.00 | -0.40 | 0.00 | -0.27 | 0.01 | -0.48 | 0.00 | 0.09 | 0.40 |
| **LDL** | -0.16 | 0.12 | 0.24 | 0.18 | 0.15 | 0.13 | 0.18 | 0.08 | -0.12 | 0.24 |
| **NAS** | 0.89 | 0.00 | 0.92 | 0.00 | 0.88 | 0.00 | 0.84 | 0.00 | -0.93 | 0.00 |
| **Steatosis** | 0.29 | 0.01 | 0.24 | 0.04 | 0.27 | 0.02 | 0.06 | 0.02 | -0.45 | 0.00 |
| **Lobular inflammation** | 0.06 | 0.03 | 0.17 | 0.04 | 0.15 | 0.02 | 0.13 | 0.02 | -0.36 | 0.00 |
| **Ballooing** | 0.15 | 0.02 | 0.23 | 0.05 | 0.30 | 0.01 | 0.31 | 0.01 | -0.24 | 0.03 |
| **Periportal inflammation** | 0.11 | 0.02 | 0.11 | 0.04 | 0.07 | 0.03 | 0.04 | 0.01 | -0.17 | 0.04 |
| **Fibrosis** | 0.28 | 0.02 | 0.37 | 0.00 | 0.22 | 0.03 | 0.02 | 0.04 | -0.15 | 0.04 |

Serum levels of CK-18-M30, IL-1Ra, FGF-21, PEDF and OPG was highly correlated with clinical characteristics, pathological features and NAS scores.
