# Supplementary material for: Combined Serum Biomarkers in Non-Invasive Diagnosis of Non-Alcoholic Steatohepatitis
Source: PLoS One. 2015 Jun 29;10(6):e0131664. doi: 10.1371/journal.pone.0131664 (PMC4486729; doi:10.1371/journal.pone.0131664)
Supplement: S4 Table — (S4 Table, DOC) (DOC) [file pone.0131664.s006.doc]

**S4 Table. Diagnostic performances of Logistic models for NASH diagnosis in NAFLD training group and validation group.**

|  | **Group** | **AUROC** | **95% CI** | **Sensitivity** | **Specificity** | **PPV** | **NPV** |
| --- | --- | --- | --- | --- | --- | --- | --- |
| **Logistic Y1** | Training group | 0.533 | 0.237-0.361 | 43% | 56% | 69% | 71% |
|  | Validation group | 0.562 | 0.301-0.422 | 42% | 51% | 67% | 69% |
| **Logistic Y2** | Training group | 0.542 | 0.198-0.305 | 46% | 53% | 66% | 68% |
|  | Validation group | 0.536 | 0.203-0.326 | 43% | 52% | 65% | 66% |

There was no statistical difference between training group and validation group (*P* >0.05).
